# Epidemiology, clinical outcomes and risk factors of third-generation cephalosporin-resistant *Escherichia coli* hospitalized infections in remote Australia—a case–control study

**DOI:** 10.1093/jacamr/dlad138

**Published:** 2023-12-19

**Authors:** Shayne Camilleri, Danny Tsai, Freya Langham, Shahid Ullah, Fabian Chiong

**Affiliations:** Department of Medicine, Alice Springs Hospital, Alice Springs, NT, Australia; Department of Infectious Diseases, Austin Health, Melbourne, VIC, Australia; Flinders Health and Medical Research Institute, Flinders University, Adelaide, SA, Australia; UQ Centre for Clinical Research, University of Queensland, Brisbane, QLD, Australia; Pharmacy Department, Alice Springs Hospital, Alice Springs, NT, Australia; Department of Medicine, Alice Springs Hospital, Alice Springs, NT, Australia; Department of Infectious Diseases, Monash Health, Melbourne, VIC, Australia; Flinders Health and Medical Research Institute, Flinders University, Adelaide, SA, Australia; College of Medicine and Public Health, Flinders University, Adelaide, SA, Australia; Department of Medicine, Alice Springs Hospital, Alice Springs, NT, Australia; Department of Infectious Diseases, Canberra Hospital, Canberra, ACT, Australia

## Abstract

**Background:**

Incidence of third-generation cephalosporin-resistant (3GCR) *Escherichia coli* infections has increased in remote Australia from 2012 to 2018.

**Objectives:**

To describe the epidemiology of 3GCR *E. coli* in Central Australia.

**Methods:**

A case–control study was conducted in the primary Central Australian hospital. Patient characteristics, antibiotic usage and clinical outcomes were compared between adult hospitalizations with 3GCR and susceptible *E. coli* isolates in 2018–19. Poisson regression was used to compare the incidence of 3GCR hospitalizations between Indigenous and non-Indigenous individuals. Patient characteristics and antibiotic usage were tested for associations with 3GCR isolates using univariate analysis.

**Results:**

A total of 889 *E. coli* isolates were identified, of which 187 (21%) were 3GCR. The incidence of 3GCR *E. coli* infection was 2.15 per 1000 person-years, with an incidence rate ratio of 6.8 (95% CI 4.6–10.1) between Indigenous and non-Indigenous individuals. When compared with the control group, 3GCR *E. coli* infections were associated with a higher Charlson comorbidity index (CCI ≥3 in 30.7% versus 15.0%, *P* < 0.001) and were more commonly healthcare associated (52.4% versus 26.7%, *P* < 0.001). A higher 1 year mortality was observed in the 3GCR group after adjustment for comorbidity (OR = 4.43, *P* = 0.002), but not at 30 days (2.4% versus 0.0%, *P* = 0.2). The 3GCR group used more antibiotics in the past 3 months (OR = 5.75, *P* < 0.001) and 12 months (OR = 3.65, *P* < 0.001).

**Conclusions:**

3GCR *E. coli* infections in remote Australia disproportionally affect Indigenous peoples and are associated with a high burden of comorbidities and antibiotic use. Strategies to enhance antimicrobial stewardship should be considered in this remote setting.

## Introduction

Antimicrobial resistance (AMR) in Enterobacteriaceae is an increasing problem, particularly third-generation cephalosporin resistance (3GCR) in *Escherichia coli*.^1^ The production of ESBL enzymes accounts for the majority of 3GCR phenotype in *E. coli.*^2^ ESBL enzymes hydrolyse many first-line β-lactam antibiotics including penicillins and most cephalosporins.^1^ First occurring as a hospital-acquired infection, ESBL-producing organisms are increasingly community acquired.^3^

Compared with third-generation cephalosporin-susceptible (3GCS) *E. coli*, 3GCR infections are often associated with worse clinical outcomes including increased mortality, treatment failure, longer length of hospital stay, and delay to effective antibiotic therapy.^1,4^ Consequently, 3GCR infections utilize greater healthcare resources and have a greater economic impact.^4^ Reported risk factors for acquiring a 3GCR infection varies in the current literature, which is likely due to differences between study populations and in local epidemiology.^5^ In general, they include previous antibiotic use, prolonged or recurrent hospitalization, and the presence of multiple comorbid conditions.^1,6^ Globally, the incidence of 3GCR *E. coli* infections ranges from less than 10% to greater than 50% of all *E. coli* infections, depending on the population.^5,7^ Nevertheless, the reported incidence is increasing across all populations.^1,5,8^ The overall incidence of 3GCR *E. coli* infections in Australia has increased from 5% in 2002 to 14.7% in 2020.^9,10^

In the Northern Territory, 23.4% of all *E. coli* blood culture isolates were 3GCR,^10^ with the incidence of 3GCR infections increasing at a greater rate in the remote Central Australian region than the national rate.^9^ Central Australia is a large and sparsely populated region (approximately 1.6 million km^2^) spanning across the southern part of Northern Territory, northern part of South Australia and eastern part of Western Australia. The region is categorized as remote or very remote. People living in rural and remote areas are more likely to experience poorer health outcomes compared with those living in urban areas due to reasons such as geographical isolation, fewer healthcare staff and resources and limited clinical decision support and surveillance systems.^11,12^ Approximately half of Central Australia’s population is Indigenous. They are some of the nation’s most geographically dispersed and socioeconomically disadvantaged population.^13^ While the incidence of 3GCR infections is growing, its epidemiology has not been studied in the remote Australian population. Understanding the characteristics and epidemiology of 3GCR *E. coli* infections has important implications for treatment decisions and improving clinical outcomes. In this study, we aimed to describe the epidemiology, identify the associated risk factors, and characterize the clinical outcomes of hospitalized 3GCR *E. coli* infections in remote Central Australia.

## Patients and methods

### Ethics

This project was approved by the Central Australian Human Research Ethics Committee (HREC Reference Number: CA-20-3723). This project was conducted in accordance with the Declaration of Helsinki and national and institutional standards.

### Study setting, design and population

A retrospective case–control study was conducted at the 210 bed primary referral hospital in Central Australia. The hospital has a large but sparsely populated catchment area, with a population of approximately 43 000 and more than 50 remote communities and outstations.

All adult hospital inpatients who cultured a 3GCR *E. coli* between 1 January 2018 and 31 December 2019 were included in the study. Patients with 3GCS *E. coli* isolates from the same period were randomly selected for a 1:1 control group. Patients were excluded if the medical records were incomplete.

**Figure 1.**
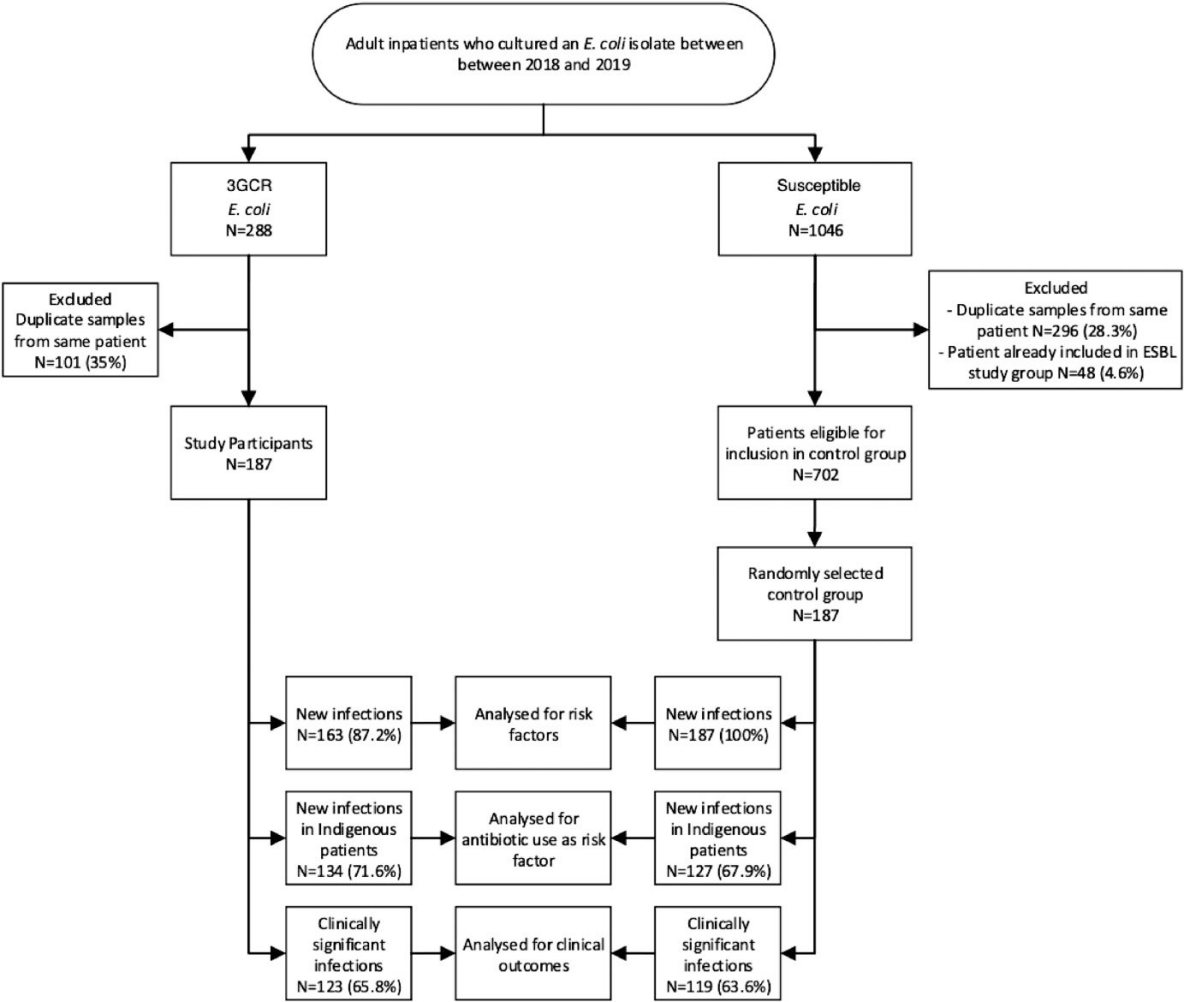
Flow chart of study design.

A three-part analysis was conducted (Figure 1). First, incidence was calculated for 3GCR *E. coli* isolates in Central Australia using population data.^13^ Second, risk factors associated with 3GCR *E. coli* were evaluated by comparing patients who had a 3GCR *E. coli* isolate for the first time with the control group. As a separate analysis, antibiotic use as a risk factor was evaluated by comparing Indigenous patients who cultured a 3GCR *E. coli* isolate for the first time with Indigenous patients in the control group. Complete antibiotic usage data for non-Indigenous patients were not available due to the majority of outpatient care occurring in the private sector in this patient group. Third, differences in outcomes between patients with clinically significant 3GCR *E. coli* infections and control group patients with clinically significant infections were compared.

### Variables and definitions

Patient data were collected from laboratory and patient electronic medical records. Demographic and clinical characteristics were collected, including prior admission to hospital, prior antimicrobial use in the 3 and 12 months prior to admission, if the isolate was hospital/community acquired or healthcare associated, clinical significance of the isolate, specimen type, diagnosis, Charlson comorbidity index (CCI)^14^ and comorbidities. Place of residence was classified as Northern Territory urban areas, remote community or other. An infection was hospital acquired or healthcare associated as defined by Friedman *et al*.^15^ The clinical significance of an isolate was determined by the treating clinicians as documented in the medical record. An isolate was considered to be a clinically significant infection if it was documented as such in the patient’s discharge summary and the patient received empirical or directed antimicrobial therapy for the infection.

The identification of *E. coli* isolates and their antimicrobial susceptibility were determined using the VITEK automated microbiology bacterial identification system (bioMérieux Inc. Marcy l’Étoile, France). The MIC was measured and interpreted as per the CLSI guidelines.^16^ 3GCR was defined as the non-susceptibility of an *E. coli* isolate to ceftriaxone and/or ceftazidime. Isolates that were not 3GCR were considered to be susceptible isolates (3GCS).

### Outcomes

The following clinical outcomes were evaluated: all-cause mortality at 30 days and 1 year, length of hospital stay, admission to the ICU, and the number of readmissions to hospital in the following 30 days and 1 year. Length of stay was defined as the number of days from admission to discharge.

### Statistical analysis

All statistical analyses were conducted using Stata version 16.1. Patients’ demographic and clinical characteristics were expressed as median and IQR for skewed data. The Mann–Whitney *U*-test was used to explore differences in patients’ characteristics and outcomes between groups. Proportions were presented as percentages of the respective denominator and were compared between groups using a standard chi-squared test for association with continuity correction, where appropriate. The incidence of 3GCR *E. coli*-associated hospitalizations was calculated with a Poisson regression model using population data from the Australian Bureau of Statistics for the local government areas within the hospital’s catchment area.^13^ Results were presented as incidence rate per 1000 person-years. For risk factor investigation, univariate logistic regression analysis was conducted to estimate ORs for the associations between first time 3GCR *E. coli* and potential risk factors. ORs were also calculated for the associations between clinical outcomes and demographics. Antibiotic use prior to admission was tested between the 3GCR and control groups using negative binomial regression models.

## Results

A total of 889 *E. coli* cases were identified (84.1% female, 69.3% Indigenous), of which 21% were 3GCR. The process of patient inclusion, randomization and group allocation for further analysis is presented in Figure 1. All patient records were complete, therefore no patients were excluded due to incomplete medical records. The baseline demographics are presented in Table 1. When compared with the control group, individuals with 3GCR *E. coli* were more likely to be of Indigenous ethnicity and reside in a remote community. The overall incidence of 3GCR *E. coli* isolates in this population was 2.15 per 1000 person-years. The incidence of 3GCR *E. coli* in the Indigenous population was significantly higher when compared with the non-Indigenous population, with an incidence rate ratio of 6.8 (95% CI 4.6–10.1, *P* < 0.001).

**Table 1.** Baseline demographic characteristics for people with 3GCR *E. coli* isolates compared with 3GCS *E. coli* isolates

Characteristics	Overall *N* = 889	3GCR *E. coli* *N* = 187	3GCS *E. coli* *N* = 702	*P* value
Age (years), median (IQR)	50 (32–64)	51 (34–64)	48 (32–64)	0.4
Indigenous, *n* (%)	616 (69.3)	157 (84.0)	459 (65.4)	<0.001
Female, *n* (%)	748 (84.1)	154 (82.4)	594 (84.6)	0.5
Place of residence				0.003
NT urban area, *n* (%)	462 (52.0)	84 (44.9)	378 (53.8)	—
Remote community, *n* (%)	376 (42.3)	98 (52.4)	278 (39.6)	—
Other, *n* (%)	51 (5.7)	5 (2.7)	46 (6.6)	—
Specimen type				0.017
Urine, *n* (%)	723 (81.3)	143 (76.5)	580 (82.6)	—
Blood, *n* (%)	85 (9.6)	17 (9.1)	68 (9.7)	—
Other, *n* (%)	81 (9.1)	27 (14.4)	54 (7.7)	—

*P* value calculated using Pearson’s chi-squared test.

NT, Northern Territory.

### Risk factors

Univariate analysis showed that those with 3GCR *E. coli* for the first time were more likely to be Indigenous, live in a remote community, have been admitted to hospital within the past 12 months, have a higher CCI and have chronic kidney disease (Table 2). 3GCR *E. coli* isolates were more likely to be healthcare associated (OR 3.14, *P* < 0.001). Control group isolates were more likely to be from a urinary source when compared with those that were 3GCR (85.0% versus 75.5%, *P* = 0.03).

**Table 2.** Demographics and clinical characteristics in people with first-time 3GCR *E. coli* isolates compared with the control group

Characteristics	Overall *N* = 350	3GCR *E. coli* *N* = 163	Control group *N* = 187	OR (95% CI)	*P* value
Age (years), median (IQR)	49 (33–64)	49 (32–63)	49 (33–65)	—	—
Indigenous, *n* (%)	261 (74.9)	134 (82.2)	127 (67.9)	2.18 (1.33–3.66)	0.002
Female, *n* (%)	291 (83.1)	133 (81.6)	158 (84.5)	0.81 (0.46–1.43)	0.5
Place of residence					
NT urban area, *n* (%)	180 (51.4)	76 (46.6)	104 (55.6)	1.69 (1.11–2.60)	0.094
Remote community, *n* (%)	152 (43.4)	82 (50.3)	70 (37.4)	0.42 (0.13–1.15)	0.016
Other, *n* (%)	18 (5.1)	5 (3.1)	13 (7.0)		0.11
Prior admission within					
3 months, *n* (%)	124 (35.4)	73 (44.8)	51 (27.3)	2.16 (1.39–3.39)	<0.001
6 months, *n* (%)	162 (46.3)	92 (56.4)	70 (37.4)	2.17 (1.41–3.34)	<0.001
12 months, *n* (%)	202 (57.7)	108 (66.3)	94 (50.3)	1.94 (1.26–3.01)	0.003
Hospital-acquired infection, *n* (%)	52 (14.9)	34 (20.9)	18 (9.6)	2.47 (1.35–4.66)	0.004
Healthcare-associated infection, *n* (%)	137 (39.1)	87 (53.4)	50 (26.7)	3.14 (2.02–4.93)	<0.001
Clinically significant infection, *n* (%)	225 (64.3)	106 (65.0)	119 (63.6)	1.06 (0.69–1.65)	0.8
Specimen type					
Urine, *n* (%)	282 (80.6)	123 (75.5)	159 (85.0)	0.54 (0.31–0.92)	0.025
Blood, *n* (%)	30 (8.6)	15 (9.2)	15 (8.0)	1.16 (0.55–2.47)	0.7
Other, *n* (%)	38 (10.9)	25 (15.3)	13 (7.0)	2.42 (1.22–5.05)	0.014
Diagnosis					
Cystitis, *n* (%)	133 (38.0)	57 (35.0)	76 (40.6)	0.79 (0.51–1.21)	0.3
Pyelonephritis, *n* (%)	28 (8.0)	14 (8.6)	14 (7.5)	1.16 (0.53–2.53)	0.7
Bacteraemia, *n* (%)	28 (8.0)	8 (4.9)	20 (10.7)	0.43 (0.17–0.97)	0.052
Asymptomatic bacteriuria, *n* (%)	105 (30.0)	50 (30.7)	55 (29.4)	1.06 (0.67–1.68)	0.8
Other, *n* (%)	56 (16.0)	34 (20.9)	22 (11.8)	1.98 (1.11–3.58)	0.022
CCI					
Median (IQR)	1 (0–2)	2 (0–4)	0 (0–2)	—	—
Individuals with index ≥3, *n* (%)	78 (22.3)	50 (30.7)	28 (15.0)	2.51 (1.50–4.28)	<0.001
Comorbidities					
Chronic kidney disease, *n* (%)	144 (41.1)	78 (47.9)	66 (35.3)	1.68 (1.10–2.59)	0.018
Haemodialysis, *n* (%)	21 (6.0)	13 (8.0)	8 (4.3)	1.94 (0.80–5.02)	0.2
Diabetes, *n* (%)	156 (44.6)	76 (46.6)	80 (42.8)	1.17 (0.77–1.78)	0.5
Chronic liver disease, *n* (%)	37 (10.6)	26 (16.0)	11 (5.9)	3.04 (1.48–6.61)	0.003

OR (95% CI) calculated using negative binomial regression model. NT, Northern Territory.

### Antibiotic use

The association between prior antibiotic use and 3GCR *E. coli* is presented in Table 3. A higher rate of antibiotic use was found in the 3GCR group when compared with the control group in the 3 months (74.6% versus 33.9%, *P* < 0.001) and 12 months (88.8% and 68.5% *P* < 0.001) prior to admission. The median days of antibiotic exposure were also higher for the 3GCR group for both 3 months (9.5 versus 0 days) and 12 months (25 versus 6 days).

**Table 3.** Antibiotic use prior to admission in Indigenous patients: first-time 3GCR *E. coli* isolates compared with the control group

	Overall *N* = 261	3GCR *E. coli* *N* = 134	Control group *N* = 127	*P* value	Incidence rate ratio (95% CI, *P* value)
Number of antibiotic use days per patient within past, median (IQR)					
3 months	2.5 (0–15)	9.5 (0–25)	0 (0–3)	<0.001	—
12 months	14 (2–40)	25 (10–62)	6 (0–21)	<0.001	—
Antibiotic use within past					
3 months, *n* (%)	143 (54.8)	100 (74.6)	43 (33.9)	<0.001	5.75 (3.40–9.92, *P* < 0.001)
12 months, *n* (%)	206 (78.9)	119 (88.8)	87 (68.5)	<0.001	3.65 (1.93–7.21, *P* < 0.001)
Antibiotic use within past 3 months by antibiotic, *n* (%)					
Penicillins	68 (26.1)	49 (36.6)	19 (15.0)	<0.001	1.90 (0.74–4.83, *P* = 0.2)
Amoxicillin and clavulanic acid	42 (16.1)	30 (22.4)	12 (9.4)	0.004	2.46 (0.90–6.65. *P* = 0.073)
Piperacillin and tazobactam	12 (4.6)	10 (7.5)	2 (1.6)	0.023	28.0 (4.12–260, *P* = 0.001)
Cephalosporin, first generation	41 (15.7)	33 (24.6)	8 (6.3)	<0.001	4.24 (1.57–11.4, *P* = 0.004)
Cephalosporin, third generation	42 (16.1)	37 (27.6)	5 (3.9)	<0.001	14.7 (5.93–38.4, *P* < 0.001)
Carbapenems	8 (3.1)	8 (6.0)	0	0.007	—
Ciprofloxacin	12 (4.6)	12 (9.0)	0	<0.001	—
Aminoglycosides	7 (2.7)	3 (2.2)	4 (3.1)	0.7	—
Doxycycline	16 (6.1)	15 (11.2)	1 (0.8)	<0.001	56.9 (9.41–489, *P* < 0.001)
Trimethoprim ± sulfamethoxazole	30 (11.5)	20 (14.9)	10 (7.9)	0.074	—
Azithromycin	20 (7.7)	13 (9.7)	7 (5.5)	0.2	2.67 (0.85–8.58, *P* = 0.093)
Antibiotic use within past 12 months by antibiotic, *n* (%)					
Penicillins	123 (47.1)	75 (56.0)	48 (37.8)	0.003	1.49 (0.76–2.92, *P* = 0.2)
Amoxicillin and clavulanic acid	78 (29.9)	46 (34.3)	32 (25.5)	0.11	—
Piperacillin and tazobactam	23 (8.8)	16 (11.9)	7 (5.5)	0.067	—
Cephalosporin, first generation	83 (31.8)	52 (38.8)	31 (24.4)	0.013	2.31 (1.19–4.48, *P* = 0.013)
Cephalosporin, third generation	79 (30.3)	57 (42.5)	22 (17.3)	<0.001	4.24 (2.30–7.85, *P* < 0.001)
Carbapenems	11 (4.2)	10 (7.5)	1 (0.8)	0.007	2.73 (0.32–22.7, *P* = 0.3)
Ciprofloxacin	23 (8.8)	19 (14.2)	4 (3.1)	0.002	27.1 (5.07–143, *P* < 0.001)
Aminoglycosides	11 (4.2)	5 (3.7)	6 (4.7)	0.7	—
Doxycycline	41 (15.7)	29 (21.6)	12 (9.4)	0.007	3.79 (1.38–10.3, *P* = 0.008)
Trimethoprim ± sulfamethoxazole	53 (20.3)	36 (26.9)	17 (13.4)	0.007	3.02 (1.07–8.44, *P* = 0.032)
Azithromycin	52 (19.2)	33 (24.6)	19 (15.0)	0.051	1.97 (1.00–3.90, *P* = 0.049)

*P* value calculated using Pearson’s chi-squared test. Incidence rate ratio (95% CI) calculated using negative binomial regression model.

### Clinical outcomes

A total of 123 (65.8%) 3GCR *E. coli* cases and 119 (63.6%) of the control group were deemed to have a clinically significant infection. The clinical outcomes of these patients are presented in Table 4. 3GCR *E. coli* infections were associated with a higher all-cause mortality at 1 year (OR 4.43, 1.72–13.7, *P* = 0.004) but not at 30 days. This association remained after adjusting for CCI (OR 3.33, 1.21–10.8, *P* = 0.03). 3GCR *E. coli* infections were also associated with a longer hospital length of stay and an increased number of readmissions at 30 days and 1 year. There were no associations between 3GCR *E. coli* infection and the need for admission to ICU.

**Table 4.** Clinical outcomes of 3GCR *E. coli* infections compared with the control group

	Overall *N* = 242	3GCR *E. coli* *N* = 123	Control group *N* = 119	*P* value	OR (95% CI, *P* value)
Length of hospital stay (days), median (IQR)	4 (1–8)	5 (1–14)	3 (0–5)	<0.001	—
Admission to ICU, *n* (%)	27 (11.2)	15 (12.2)	12 (10.1)	0.6	—
Death *n* (%)					
At 30 days	3 (1.2)	3 (2.4)	0 (0)	0.2	—
At 1 year	25 (10.3)	20 (16.3)	5 (4.2)	0.002	4.43 (1.72–13.7, *P* = 0.004)
At 1 year adjusted for CCI					3.33 (1.21–10.8, *P* = 0.028)
Readmissions, *n* (%)					
≥1 readmission within 30 days	46 (19.0)	33 (26.8)	13 (10.9)	0.002	2.99 (1.51–6.21, *P* = 0.002)
≥3 readmissions within 1 year	58 (24.0)	38 (30.9)	20 (16.8)	0.010	2.21 (1.21–4.15, *P* = 0.011)

*P* value calculated using Pearson’s chi-squared test. OR (95% CI) calculated using negative binomial regression model.

## Discussion

To the best of our knowledge, this is the first study describing the epidemiology of 3GCR *E. coli* in Central Australia. We have characterized the high burden and impact of 3GCR *E. coli* in this setting. Indigenous peoples are disproportionately affected, accounting for 82.2% of all patients with 3GCR *E. coli*, while making up only 43.4% of the Central Australian population.^13^ We have identified associations of this high incidence rate with residence in a remote community, high burden of comorbidity (especially chronic kidney and liver diseases), frequency of antibiotic use and hospital admissions.

In our study, 21% of all *E. coli* isolates identified were 3GCR. This is compared with the average incidence of 14.7% across Australia,^10^ 27.7% in Eastern Europe and 33.9% in Latin America.^7^ Unique to Central Australia is the high rate of Indigenous residents, and a geographically dispersed and remote-living population. Our study cohort, being predominantly Indigenous, had a high burden of chronic disease. A third of patients in the 3GCR group had a CCI >3. Indigenous Australians are known to experience a higher burden of chronic disease, at 2.3 times that of non-Indigenous Australians, and have a shorter life expectancy.^17,18^ Life expectancy and measures of most health metrics have also been reported as poorer for Indigenous Australians residing in remote locations when compared with non-remote locations.^17^

The risk factors for 3GCR *E. coli* identified in this study were similar to previously reported data. They include higher rates of comorbidities, antibiotic use and recent and frequent hospitalization.^6,19^ While there were significantly more hospital-acquired isolates in the 3GCR group, the majority of 3GCR *E. coli* isolates in this study were not hospital acquired. This is consistent with worldwide changes in epidemiology, in which 3GCR *E. coli* are increasingly community acquired.^3^ In our study, over half of the 3GCR *E. coli* isolates were healthcare associated (87; 53.4%), which indicates a highly comorbid population with frequent healthcare needs.

Increased antibiotic use, misuse and overuse are known to be risk factors for the development of AMR due to the selective pressure.^12,20^ Antibiotic usage was significantly higher in our study population than in the general population.^9^ In the 3GCR group, 88.8% had at least one antibiotic in the past 12 months compared with 68.5% of the control group, and 40.3% of the Australian population.^9^ The high use of antibiotics in our study population could be a contributor to the increased rates of 3GCR *E. coli* observed in Central Australia.^12,20^ A high burden of infections and chronic diseases, housing overcrowding and complex sociodemographic factors likely all contribute to higher antibiotic use in this population.^21,22^ The geographically dispersed and remote location also impacts antibiotic use, with rurality shown to be a risk factor for inappropriate antibiotic prescribing and antibiotic overuse.^12^ In conjunction with addressing the complex sociodemographic factors that contribute to an increased burden of infections, the implementation of a comprehensive antimicrobial stewardship programme (including education, clinical support, surveillance and polices) would help reduce antibiotic overuse and improve the appropriateness of antibiotic prescribing in rural and remote communities.^12,21^

In our study, 3GCR *E. coli* infections were not associated with 30 day mortality, albeit with a low mortality rate observed in both groups (2.2% 3GCR versus 0.0% control group, *P* = 0.20). The younger age of our study population may have compensated for the propensity to poor acute prognosis identified in other studies.^4,6^ The mean age of our study cohort was consistent with others in the literature focusing on Indigenous participants, which ranges between 40 and 49 years compared with >60 years in other populations.^6,23–25^ 3GCR *E. coli* infections were associated with a higher mortality rate at 1 year (OR = 4.43, *P* = 0.004), even after adjustment for comorbidity (OR = 3.33, *P* = 0.028). We also found an association between 3GCR infections and longer hospital admissions (5 versus 3 days, *P* > 0.001) and multiple hospital readmission within 1 year (OR 2.21, *P* = 0.011), which further demonstrates poorer health outcomes for an already disadvantaged and vulnerable population.

A major strength of this study is the comprehensive dataset. As the only hospital serving a very large and remote population, every Central Australian resident admitted to hospital who cultured *E. coli* during the study time frame was captured for detailed analysis. This study has several limitations. Firstly, while being a single-centre study allowed us to better characterize 3GCR *E. coli* in our population, it reduces the external validity, meaning our results may not be generalizable to other populations. Small patient numbers limited the power of our analysis and the ability to analyse specific associations using multivariate analysis. Secondly, community antibiotic prescribing data were only available for Indigenous patients. This accounted for the majority of patients in the study; however, it did limit the ability to assess for antibiotic use as a risk factor across the whole study population. Lastly, patient-specific socioeconomic and housing data were not available for statistical testing of correlations. Future studies could examine patient-specific socioeconomic and housing data to comprehensively characterize the factors that contribute to the increased rates of 3GCR *E. coli* in this population.

In conclusion, there is a high incidence of 3GCR *E. coli* in remote Central Australia, which disproportionately affects Indigenous Australians. A high burden of comorbidities, increased rates of antibiotic use, and recent and frequent hospitalization were associated with 3GCR *E. coli* isolates. 3GCR *E. coli* infections were associated with poorer clinical outcomes and 1 year mortality. Strategies to improve antimicrobial stewardship in the remote setting are warranted to reduce the incidence of 3GCR *E. coli*.
